# Accelerated Solvent Extraction of Terpenes in Cannabis Coupled With Various Injection Techniques for GC-MS Analysis

**DOI:** 10.3389/fchem.2021.619770

**Published:** 2021-04-01

**Authors:** Colton Myers, Jason S. Herrington, Paul Hamrah, Kelsey Anderson

**Affiliations:** ^1^ Restek Corporation, Bellefonte, PA, United States; ^2^ Verity Analytics, San Diego, CA, United States

**Keywords:** accelerated solvent extraction (ASE), terpenes, solid-phase microextraction (SPME), solid-phase microextraction Arrow (SPME Arrow), gas chromatography–mass spectrometry (GC-MS)

## Abstract

The cannabis market is expanding exponentially in the United States. As state-wide legalization increases, so do demands for analytical testing methodologies. One of the main tests conducted on cannabis products is the analysis for terpenes. This research focused on implementation of accelerated solvent extraction (ASE), utilizing surrogate matrix matching, and evaluation of traditional vs. more modern sample introduction techniques for analyzing terpenes via gas chromatography–mass spectrometry (GC-MS). Introduction techniques included Headspace-Syringe (HS-Syringe), HS-Solid Phase Microextraction Arrow (HS-SPME Arrow), Direct Immersion-SPME Arrow (DI-SPME Arrow), and Liquid Injection-Syringe (LI-Syringe). The LI-Syringe approach was deemed the most straightforward and robust method with terpene working ranges of 0.04–5.12 μg/mL; *r* ^2^ values of 0.988–0.996 (0.993 average); limit of quantitation values of 0.017–0.129 μg/mL (0.047 average); analytical precisions of 2.58–9.64% RSD (1.56 average); overall ASE-LI-Syringe-GC-MS method precisions of 1.73–14.6% RSD (4.97 average); and % recoveries of 84.6–98.9% (90.2 average) for the 23 terpenes of interest. Sample workflows and results are discussed, with an evaluation of the advantages/limitations of each approach and opportunities for future work.

## Introduction

The legal cannabis market is one of the fastest growing markets across the globe. In 2019, cannabis use for medicinal purposes in the United States generated $4 billion to $4.9 billion in sales, compared to the adult-use estimates between $6.6 billion and $8.1 billion (Marijuana Business Daily, 2020). As the United States and additional countries continue to legalize the use of medicinal and recreational cannabis, analytical testing demands increase. A 2020 report by Market Data Forecast valued the global cannabis testing market at $1,218.0 million in 2019 and estimated it to be growing at a compound annual growth rate (CAGR) of 12.42% (Market Data Forecast, 2020). The market is projected to almost double at $2,187.3 million by 2024 (Market Data Forecast, 2020). Of the examinations conducted in cannabis testing laboratories, terpenes profiling is a popular analysis, regardless of state regulations. Terpenes are a naturally occurring set of organic compounds, which are commonly found in plants, and are typically strong in odor (Nutinnen, 2018). Terpenes are made up of isoprene units and are classified by the number of their isoprene units (Nutinnen, 2018). The two types of terpenes that are commonly analyzed in the cannabis testing industry are monoterpenes, which have two isoprene units, and sesquiterpenes, which have three isoprene units. Over 100 terpenes have been identified in different cannabis chemical varieties (chemovars) (Calvi et al., 2018). Each cannabis chemovar has its own unique terpene profile giving consumers different aromas, flavors, and experiences depending on the chemovar they use. According to Russo et al., terpenes play a major role in the entourage effect, which is the synergistic interaction between phytocannabinoids and terpenoids with respect to treating numerous ailments (e.g., depression) (Russo, 2011). The desire to understand and capitalize on this entourage effect is the motivation for terpenes testing in the cannabis industry.

Terpenes have been analyzed in numerous commodities within the food and beverage industry. Previous studies have looked at a variety of matrices (e.g., tequila) and have used different analytical techniques [e.g., solid phase microextraction (SPME)] to conduct the analysis (Abilleira et al., 2010; Kupska et al., 2014; Cacho et al., 2015; Jelen and Gracka, 2015; Stenerson and Halpenny, 2017; Calvi et al., 2018; Brown et al., 2019; Ibrahim et al., 2019; Shapira et al., 2019; Bakro et al., 2020; Gaggiotti et al., 2020; Muñoz-Redondo et al., 2020; Nguyen et al., 2020; Ternelli et al., 2020; Zhang et al., 2020). However, only a few studies have shown the analysis of terpenes in cannabis/hemp matrices (e.g., flower, gummy), and their robustness for compliance laboratories remains uncertain. Calvi et al., Ternelli et al., Gaggotti et al., and Stenerson et al. did not perform extractions on cannabis/hemp samples; rather they added the samples directly to a headspace (HS) vial and demonstrated the analysis of terpenes using HS-SPME (Stenerson and Halpenny, 2017; Calvi et al., 2018; Gaggiotti et al., 2020; Ternelli et al., 2020). Nguyen et al. utilized a pseudo extraction by adding a solvent to dried material, followed by analysis via HS-GC-MS (Nguyen et al., 2020). The five aforementioned studies appear to lack an exhaustive cannabis/hemp extraction, and therefore this calls into question the real-world applicability of these. Furthermore, Calvi et al., Ternelli et al., Gaggotti et al., and Stenerson et al. only focused on profiling the terpenes in the cannabis/hemp matrices studied and therefore only presented qualitative and semiquantitative data.

Bakro et al., Brown et al., Ibrahim et al., and Shapira et al. extracted cannabis flower with ethanol, hexane, ethyl acetate, and methanol, respectively, and provided quantitative results (Brown et al., 2019; Ibrahim et al., 2019; Shapira et al., 2019; Bakro et al., 2020). However, Bakro et al. only looked at hemp and used a nonspecific GC-FID approach, which is cumbersome when attempting to differentiate between coeluting terpenes of interest and matrix interferences. Brown et al. did not provide method accuracies for all targeted terpenes and reported less than desirable linearities, which fell below an *r* ^2^ value of 0.960 for each terpene of interest (Brown et al., 2019).

To date, the most promising methods presented by Ibrahim et al. and Shapira et al. utilized exhaustive cannabis/hemp extraction approaches followed by GC-MS and reported desirable quantitative results (Ibrahim et al., 2019; Shapira et al., 2019). Ibrahim et al. and Shapira et al. used sample introduction techniques like liquid injection without filtration and SHS-GC-MS, respectively. More importantly, none of the aforementioned studies accounted for matrix effects, as they all used solvent-based calibrations and, due to the complexity and dirtiness of cannabis matrices, this could lead to inaccurate reporting (Raposo and Barceló, 2020). In addition, these studies did not evaluate more modern sample extraction approaches [e.g., accelerated solvent extraction (ASE)] and/or sample introduction techniques (e.g., DI-SPME Arrow).

The following study was conducted to evaluate more modern sample preparation and introduction techniques and demonstrate their potential value to cannabis compliance testing laboratories in need of guidance for qualitative and quantitative terpenes analysis. In addition, this study evaluated accelerated solvent extraction (ASE 350) of terpenes in cannabis samples, which is commonly used in other markets within the analytical testing industry (Ligor et al., 2014; Chiesa et al., 2017; Hu et al., 2020; Ning et al., 2020). Furthermore, to avoid potentially inaccurate reporting, matrix matched standards were used for calibration. Finally, the more traditional Headspace-Syringe (HS-Syringe) and Liquid Injection-Syringe (LI-Syringe) approaches were compared to the more modern HS-Solid Phase Microextraction Arrow (HS-SPME Arrow) and Direct Immersion-SPME Arrow (DI-SPME Arrow), which has recently demonstrated enhanced robustness and improved sensitivity over traditional SPME fibers (Herrington et al., 2020).

## Experimental

The following experimental sections describe the detailed procedures utilized during the three main parts of this manuscript: 1. *HS-Syringe vs. HS-SPME Arrow vs. DI-SPME Arrow* for the determination of the preferred sample introduction approach with the use of terpenes in solution; 2. *Terpene Extraction Evaluation* for the evaluation of an ideal terpenes extraction method for cannabis flower; 3. The information gathered in *HS-Syringe vs. HS-SPME Arrow vs. DI-SPME Arrow* and *Terpene Extraction Evaluation* were then combined for a final comparison with an existing validated LI-Syringe technique [i.e., validated with the California Bureau of Cannabis Control (BCC)] outlined in *DI-SPME Arrow vs. LI-Syringe*.

### Materials and Reagents

Hop pellets were obtained from the Beverage Factory (San Diego, CA, United States) and stored at −10 °C for 1 h. Dried cannabis material was obtained from Cream of the Crop (San Diego, CA, Unites States) and stored at −10 °C for 1 h. Cannabis Terpene Standards #1 and #2 (cat# 34095 and 34096) were purchased from Restek Corporation (Bellefonte, PA, United States). Napthalene-d8 was purchased from Restek Corporation (Bellefonte, PA, United States). Isopropanol (IPA) was purchased from Filtrous (LCMS Grade) and 1.1 mm, 120 µm DVB/PDMS SPME Arrows were purchased from Restek Corporation (Bellefonte, PA, United States).

### HS-Syringe vs. HS-SPME Arrow vs. DI-SPME Arrow

The following experiments were conducted to evaluate the differences between HS-Syringe, HS-SPME Arrow, and DI-SPME Arrow as sample introduction techniques. Each technique was evaluated using Cannabis Terpene Standard #1 and #2 (cat# 34095 and 34096) from Restek Corporation (Bellefonte, PA, United States). Samples were evaluated using the same GC-MS conditions shown in Supplementary Table S1. For the HS-Syringe and HS-SPME Arrow samples, a standard stock solution was made by diluting both standards into one solution for a final concentration of 5 μg/mL in IPA. Samples were prepared by adding 1.5 g of NaCl to a 20 mL HS vial, followed by 1 mL of 5 μg/mL stock solution and 4 mL of water for a final concentration of 1 μg/mL (see sampling conditions in Supplementary Tables S2, S3). Previous work (results not shown) demonstrated that HS-SPME Arrow analyte responses were higher and more reproducible when using an incubation temperature of 40 °C or less, hence having lower incubation temperature compared to the HS-Syringe method. The DI-SPME Arrow samples were prepared by diluting both standards into one stock solution for a final concentration of 20 μg/mL. To a 20 mL HS vial, 1 mL of the 20 μg/mL stock solution was added, followed by 19 mL of water for a final concentration of 1 μg/mL (same concentration for HS-Syringe and HS-SPME Arrow, see sampling conditions in Supplementary Table S3). Each technique was run in triplicate for the initial evaluation of sample introduction techniques.

### Terpene Extraction Evaluation

An evaluation of terpene extraction processes was conducted to understand advantages and limitations of certain techniques. Extractions using the Dionex Accelerated Solvent Extractor (ASE 350) were compared to a hand-solvent extraction for terpene analysis. Three chemical varieties (chemovars) were used to evaluate both extraction techniques. Cannabis flower was frozen at −10 °C for 1 h then homogenized on a sheet pan with a rolling pin. For ASE 350 extractions, 0.5 g of homogenized cannabis flower was weighed and added to a 10 mL stainless steel ASE 350 cell and the remaining cell volume was lightly packed with diatomaceous earth. The cell was then extracted using the parameters in Table 1. The extract was then diluted to 12 mL in a graduated cylinder due to convenience and because this approach achieved the desired data quality objectives of this study (e.g., method precision RSDs <15%). However, future researchers may consider the use of volumetric flasks to achieve better precision. 1 mL of the cannabis flower extract was added to a 2.5 mL autosampler vial and then analyzed. For hand extractions, 0.5 g of homogenized cannabis flower was weighed and added to a 50 mL centrifuge tube, followed by 12 mL of IPA. Samples were vortexed for 3 min and sonicated at 40 °C for 5 min. Samples were then centrifuged in a Sorvall RT7 Plus centrifuge for 3 min. 1 mL of the supernatant was added to a 2.5 mL autosampler vial and then analyzed. ASE 350 and hand extractions were analyzed via GC-FID.

**TABLE 1:** Accelerated solvent extractor (ASE) parameters for extracting terpenes from hops pellets and cannabis flower.

Dionex ASE 350	
Temperature	75 °C
Pressure	1500 psi
Extraction solvent	Isopropanol (IPA)
Static time	5 min
Purge time	90 s
Heat time	5 min
Cycles	1

### DI-SPME Arrow vs. LI-Syringe

Results from the experiments outlined in *HS-Syringe vs. HS-SPME Arrow vs. DI-SPME Arrow* and *Terpene Extraction Evaluation *indicated DI-SPME Arrow was the preferred sample introduction approach, and ASE was the ideal terpene extraction technique for cannabis. This information was then utilized for a comparison to an existing validated LI-Syringe method. However, the experiments conducted in *HS-Syringe vs. HS-SPME Arrow vs. DI-SPME Arrow* was carried out in Pennsylvania, and the use of cannabis flower necessitated a fully licensed laboratory, which was located in California. The DI-SPME Arrow parameters outlined in Table 2 had been further optimized for terpenes analysis in cannabis and used in the California laboratory; however, they are only slightly different from the initial DI-SPME Arrow parameters outlined in Supplementary Table S3. In addition, a GC-MS/MS was used in single quad MS mode in California Laboratory, since single quad MS is more common for this analysis (see parameters in Table 3). Furthermore, a selected ion monitoring (SIM) method (Supplementary Table S4) was utilized to help eliminate background noise and provide better sensitivity. LI-Syringe was only evaluated in California using the parameters listed in Table 3.

**TABLE 2:** Optimized and final DI-SPME Arrow parameters.

CTC PAL parameters	
Tool	DI-SPME Arrow
Agitator	Agitator 1
Heatex Stirrer	Heatex Stirrer 1
Injector penetration depth	50 mm
Incubation time	1 min
Extraction time	2 min
Incubation/Extraction temperature	40 °C
Desorption time	60 s
Pre/Post conditioning	No/Yes
Conditioning time	60 s
Conditioning temperature	280 °C

**TABLE 3:** Analytical parameters for evaluating terpenes in cannabis with DI-SPME Arrow and LI-Syringe.

Thermo Scientific^TM^ Trace^TM^ 1310/TSQ^TM^ 9000 parameters	
Column	Rxi-624Sil MS - 30 m × 0.25 mm × 1.4 μm
Injection	Liquid injection (LI-Syringe)
Inj. Vol.	1 µL
Liner	Topaz 4.0 mm precision liner w/Wool [cat# 23305 (LI-Syringe)] Topaz 1.8 mm ID straight/SPME liner [cat# 23280 (DI-SPME)]
Inj. Temp.	280 °C
Purge flow	5 mL/min
Oven	80 °C (hold 0 min) to 130 °C (hold 4 min) by 20 °C/min to 275 °C (hold 0.47 min) by 17 °C/min
Carrier gas	He, constant flow
Flow rate	1.5 mL/min
Detector	MS
Mode	SIM
Transfer line temp.	275 °C
Ion source temp.	275 °C
SIM	See Supplementary Table S4

#### Hops Pellets and Cannabis Flower Preparation

Hops pellets were utilized as a terpene-free surrogate to matrix match cannabis flower for the following DI-SPME Arrow vs. LI-Syringe data: calibration curves, laboratory control samples (LCS), continuing calibration verification (CCV) samples, detection limit, and analytical precision samples. Hops were crushed and homogenized on a sheet pan with a rolling pin. The crushed hops were then cleaned with a proprietary solvent cleaning process to eliminate the presence of terpenes. Following solvent cleaning, the hops were dried in an oven. For the DI-SPME Arrow and LI-Syringe method precision experiments, cannabis shake (small pieces of cannabis flower that break off of larger buds) was homogenized and utilized. For the cannabis chemovar experiments, the flower was crushed and homogenized on a sheet pan using a rolling pin.

#### Accelerated Solvent Extractor

The following DI-SPME Arrow vs. LI-Syringe experiments were conducted utilizing hops pellets and cannabis flowers which were extracted using an ASE 350 with the parameters previously shown in Table 1. For all of the following DI-SPME Arrow and LI-Syringe experiments, either 0.5 g of cleaned hops or 0.5 g of cannabis flower was weighed out and placed into a 10 mL ASE 350 stainless steel extraction cell. Diatomaceous earth was then slowly added and lightly packed to fill the remaining volume in the cell. Samples were then extracted using IPA. Other work has shown that extracting with IPA can lead to poor peak shape for the terpenes of interest (Krill et al., 2020). However, IPA gave desirable peak shape for this study and was used because of its cost, convenience, and toxicity relative to other solvents demonstrated for cannabis extractions.

#### After ASE Processing

After ASE extraction, all extracts, which were typically between 10 and 11 mL, were brought to a final volume of 12 mL in order to consistently evaluate extracts of the same volume. Using a 3 mL Luer lock syringe with 0.22 µm filter, 3 mL of extract was filtered. For DI-SPME Arrow experiments, 1 mL of the filtered extract was added to 19 mL of LCMS grade water (i.e., 20 mL final volume) in a 20 mL headspace vial. In addition, 20 µL of 100 μg/mL internal standard (ISTD) solution was added. Subsequently, the headspace vial was capped and spinned for 10 s. For LI-Syringe experiments, 500 µL of the filtered extract was added to a 2 mL autosampler vial. In addition, 5 µL of the 10 μg/mL ISTD solution was added. Subsequently, the autosampler vial was capped and spinned for 10 s.

#### Terpenes Standards and Internal Standards

Differences in linear range between DI-SPME Arrow and LI-Syringe necessitated the use of the different intermediate and ISTD solutions. Intermediate concentrations of 1000 μg/mL and a 10 μg/mL were prepared from the 2,500 μg/mL terpene standards 1 and 2. To prepare the 1000 μg/mL intermediate, 400 µL of each terpene standard (i.e., 800 µL total) was added to 200 µL of IPA, cap and vortex. The 10 μg/mL intermediate was prepared from the 1000 μg/mL intermediate by adding 10 µL of the 1000 μg/mL intermediate to 990 µL of IPA, cap and vortex. A solution of naphthalene-d8 ISTD was made at 100 μg/mL for DI-SPME Arrow experiments and 10 μg/mL for LI-Syringe experiments.

#### DI-SPME Arrow Calibration

For the highest DI-SPME Arrow calibration point (level 7), 153.6 µL of 1000 μg/mL terpene solution was added to hops. Once extracted, the extract was brought to 12 mL with IPA and filtered, thereby reducing calibration level 7–12.8 μg/mL. Intermediate serial dilutions (Supplementary Table S5) were carried out on calibration level 7 to make the other 6 calibration points. For example, 1500 µL of calibration level 7 was added to 1500 µL of IPA to make calibration level 6. This process was then repeated for the other calibration points. However, the final calibration solutions required a secondary dilution into 20 mL headspace vials (Supplementary Table S6). For example, 1 mL of the calibration level 6 (i.e., 6.4 μg/mL) was added to 19 mL of water (i.e., 20 mL total volume) for a final concentration of 0.32 μg/mL and then spiked with ISTD. For a DI-SPME Arrow CCV equivalent to calibration level 3, calibration 7 filtered extract was diluted with IPA, spiked with ISTD, capped, and vortexed for 10 s.

#### LI-Syringe Calibration

For the highest LI-Syringe calibration point (level 10), 61.4 µL of the 1000 μg/mL terpene solution was added to the hops. Once extracted, the extract was brought to 12 mL with IPA and filtered, thereby reducing calibration level 10 to 5.12 μg/mL. Serial dilutions (Supplementary Table S7) were carried out on calibration level 10 to make the other 9 calibration points. For example, 500 µL of calibration level 10 was added to 500 µL of IPA to make calibration level 9. This process was then repeated for the other calibration points. Finally, 5 µL of the 10 μg/mL ISTD solution was added to each calibration vial at levels 2–10, and 10 µL of the 10 μg/mL ISTD solution was added to level 1 given the difference in final volume. After being spiked with ISTD, the vial was capped and spinned for 10 s. See Supplementary Table S7 for the LI-Syringe calibration curve.

#### Method Validation and Chemovar Experiments

This section addresses the following method validations: method detection limit (MDL)/limit of quantitation (LOQ), analytical precision, method precision, and % recovery. The DI-SPME Arrow and LI-Syringe MDLs/LOQs were determined from seven replicate low level calibration points. In addition, LCSs were run to determine the analytical precision and % recovery of both methods. For a DI-SPME Arrow LCS, 76.8 µL of the 1000 μg/mL intermediate terpene solution was added to hops (equivalent to calibration level 6). For an LI-Syringe LCS, 384 µL of 10 μg/mL intermediate terpene solution was added to the hops (equivalent to calibration level 6). It is important to note that the LCS represents a separate hops spike and extraction. Furthermore, the DI-SPME Arrow and LI-Syringe method precisions were determined from seven different aliquots of cannabis shake. Finally, three different chemovars of cannabis flower were evaluated for terpenes with DI-SPME Arrow and LI-Syringe.

## Results and Discussion

### HS-Syringe vs. HS-SPME Arrow vs. DI-SPME Arrow

**FIGURE 1:**
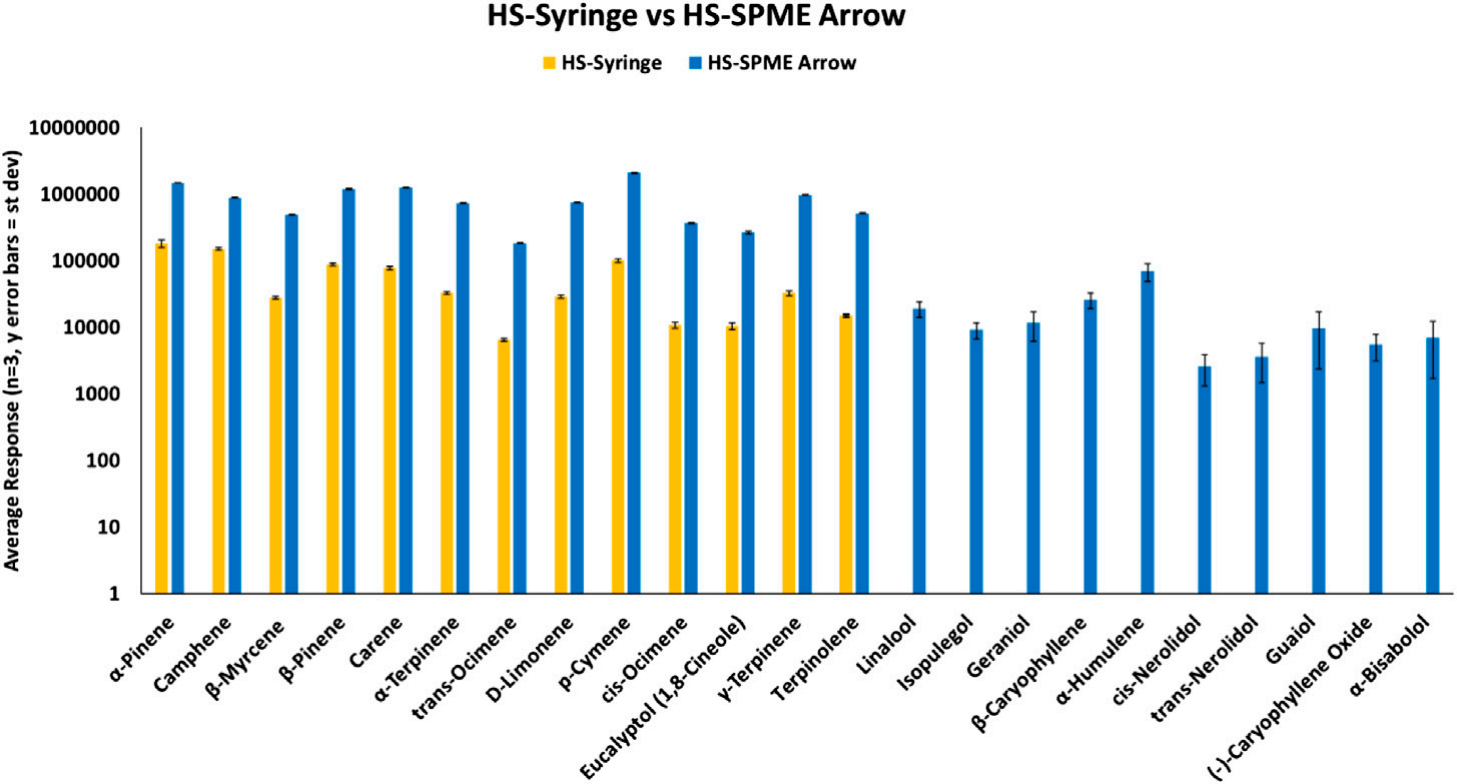
HS-SPME Arrow vs DI-SPME Arrow for terpenes.

Initial work compared three different types of sample preparation/introduction techniques for terpene analysis via GC-MS. Techniques were evaluated based on relative compound response using only reference terpene standards. First, the more traditional approach using a HS-Syringe was compared to HS-SPME Arrow (120 µm DVB/PDMS). As shown in Figure 1, 13 of 23 terpenes were identified using the HS-Syringe approach. However, this approach was unable to effectively pick-up the later eluting and less volatile terpenes, which fall into the sesquiterpene category. When samples were analyzed via HS-SPME Arrow, 23 of 23 terpenes were able to be identified.

When comparing responses for the 13 terpenes that were able to be identified in both approaches, HS-SPME Arrow had much greater responses than the HS-Syringe approach. For the terpenes found in both HS techniques, the responses on the SPME Arrow were >10× that of the HS-Syringe. Both samples were prepared identically and analyzed with the suggested parameters for each technique. When first looking at the HS-Syringe results, it was unclear if the less volatile sesquiterpenes were partitioning into the HS of the 20 mL vial. However, after analyzing the results for the HS-SPME Arrow and detecting the less volatile compounds, it was confirmed that these compounds are partitioning into the HS of the vial. It is not clear as to where the terpenes were lost (i.e., not transferred efficiently) in the HS-Syringe process, and it was outside of the scope of this study to determine the root cause. Because the HS-SPME Arrow method was able to identify all of the terpenes in the samples, this approach was chosen to move forward in the study. However, it was desired to see how HS-SPME Arrow compared to DI-SPME Arrow.

**FIGURE 2:**
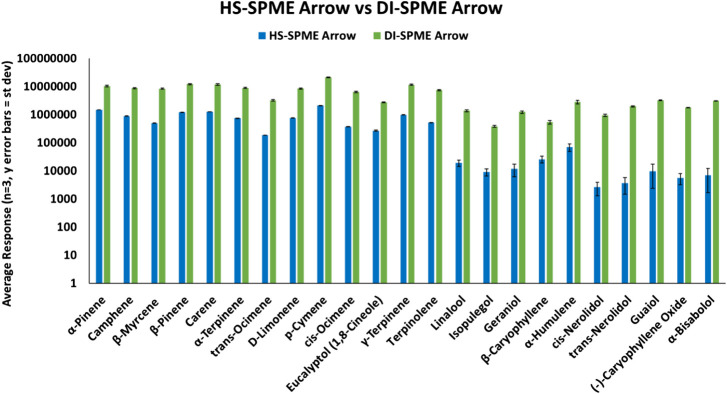
HS-SPME Arrow vs DI-SPME Arrow for terpenes.

HS-SPME Arrow samples and DI-SPME Arrow samples were prepared according to their respective approach, but were analyzed under the same instrument conditions. Both techniques were able to identify all terpenes within the reference standard samples. However, as shown in Figure 2, terpene samples analyzed via DI-SPME Arrow showed improved responses over HS-SPME Arrow, especially for the higher molecular weight terpenes and also proved to be more reproducible (i.e., better precision). Responses for the DI-SPME Arrow averaged 6× greater than that of the HS-SPME Arrow. %RSDs for the HS-SPME Arrow were as high as 76%, while all DI-SPME Arrow %RSDs were ≤15%. Potential limitations of DI-SPME Arrow include shortened fiber lifetime and/or increased matrix exposure; however, given the improved responses and reproducibility, it was selected as the technique to move forward for further method validation and compared against a traditional liquid-injection- (LI-) syringe method.

### Hand Shakeout vs. Accelerated Solvent Extractor

Several terpene extraction approaches were considered for the current study. The Full Evaporative Technique (FET), which is popular within the cannabis testing industry, was not evaluated in the current study as this technique’s foundation is HS-Syringe; and the results discussed in *HS-Syringe vs. HS-SPME Arrow vs. DI-SPME Arrow* demonstrated that HS-Syringe did not perform as well as HS-SPME Arrow, which was also inferior to DI-SPME Arrow for the analysis of terpenes. Other industries already capitalize on the benefits of ASE 350 (Ligor et al., 2014; Chiesa et al., 2017; Hu et al., 2020; Ning et al., 2020). Therefore, a simple hand shakeout solvent extraction method was compared to an ASE 350 extraction method to evaluate the performance of each technique for extracting terpenes from cannabis flower. Three different cannabis chemovars were extracted using both techniques and the average of their FID responses were determined (Table 4). Both techniques extracted the same 13 terpenes from the cannabis flower. On average, the hand shakeout responses were better than the ASE 350 responses for 11 of the 13 terpenes detected. Given the small sample size, a nonparametric Kruskal-Wallis test was completed to compare the averages and determine if there was a statistical difference between the hand shakeout and ASE 350 approaches. With the exception of Camphene and Linalool (*p* = 0.050), the Kruskal–Wallis tests indicate a general trend of no statistically significant difference between the hand shakeout and ASE 350 extraction techniques for the 13 detected terpenes.

**TABLE 4:** Hand shakeout vs. accelerated solvent extraction (ASE 350) for extraction of terpenes from cannabis flower.

Compound	Hand shakeout	ASE 350	Kruskal-Wallis
	**Average FID response factor**	**Average FID response factor**	
α-Pinene	0.055	0.044	0.127
Camphene	0.019	0.014	0.05
β-Myrcene	0.0104	0.101	0.827
β-Pinene	0.107	0.115	0.827
Carene	ND	ND	NA
α-Terpinene	ND	ND	NA
*trans*-Ocimene	ND	ND	NA
D-Limonene	0.545	0.442	0.275
*p*-Cymene	ND	ND	NA
*cis*-Ocimene	0.011	0.007	0.248
Eucalyptol (1,8-cineole)	ND	ND	NA
γ-Terpinene	ND	ND	NA
Terpinolene	ND	ND	NA
Linalool	0.241	0.176	0.050
Isopulegol	ND	ND	NA
Naphthalene-d8 (ISTD)	ND	ND	NA
Geraniol	0.019	0.023	0.248
β-Caryophyllene	1.318	1.108	0.275
α-Humulene	0.611	0.523	0.275
*cis*-Nerolidol	0.298	0.242	0.127
*trans*-Nerolidol	0.863	0.734	0.127
Guaiol	ND	ND	NA
(-)-Caryophyllene Oxide	ND	ND	NA
α-Bisabolol	14.94	6.45	0.275

NA, not applicable; ND, not detected.

In addition, several factors were considered when selecting the extraction method. The hand shakeout required more use of nonreusable consumables (e.g., centrifuge tubes), making it a “greener” approach. In addition, the cannabis flower hand shakeout technique could potentially differ depending on the lab technician completing the manually intensive hand shakeout extraction. On the contrary, the ASE 350 cells were reused by cleaning after extraction. Furthermore, the extraction consistency that the ASE 350 offers was not user dependent. The lack of statistical significance between the two extraction techniques, coupled with less consumable needs and user variability, leads to utilizing the ASE 350 for all of the following DI-SPME Arrow vs. LI-Syringe experiments. It is important to note that this was the first study to utilize an ASE 350 for the extraction of terpenes from cannabis. Despite this novel development for the field of cannabis, future studies should consider the further development of the current ASE 350 parameters to optimize extraction efficiency by changing solvent extraction ratios, extraction temperature, etc. In addition, this technique lends itself very well to joint application potential with potency and/or pesticides, therefore making it a very desirable technique (Restek, 2020).

### Hops for Clean Surrogate Matrix

An initial comparison of identical calibrations curves prepared in 100% IPA and matrix demonstrated matrix effects (MEs) (Raposo and Barceló, 2020). Of the 23 terpenes evaluated, 17 had positive MEs (7 average), as defined by Chambers et al. (2007). This observation suggested there was a “soft” signal enhancement for these compounds, which may be the result of matrix-induced chromatographic response (Hajslova and Zrostlikova, 2003). The remaining 6 terpenes (Linalool, Isopulegol, Geraniol, β-Caryophyllene, α-Humulene, and *trans*-Nerolidol) had negative MEs (-18 average), which suggested a “soft” signal suppression. The signal suppression for the aforementioned alcohols is contradictory to the theory of matrix-induced chromatographic response and represents a testament to the complexities of matrix effects. Regardless, these results indicated that cannabis flower MEs were present and therefore a matrix match calibration approach was deemed ideal. However, it was outside the scope of the current manuscript to fully dissect all of the current ME phenomenon associated with cannabis, especially considering the wide array of cannabis matrices. Future researchers are encouraged to expand upon the current start to understanding cannabis MEs.

**FIGURE 3:**
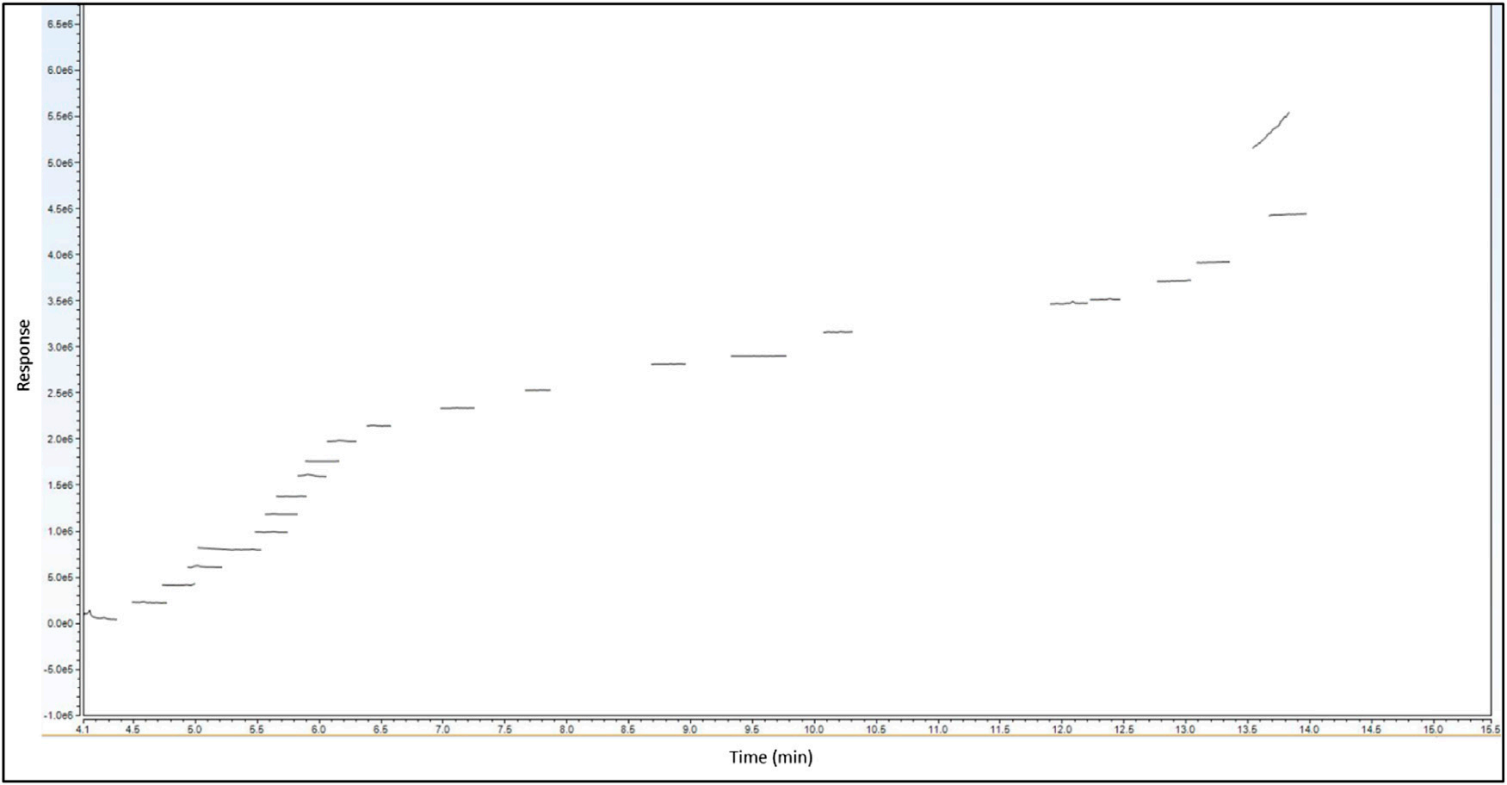
Cleaned hops blank (LI-Syringe) demonstrating terpene-free surrogate matrix.

To date, the cannabis industry has not been utilizing surrogate matrices, despite the fact that studies have demonstrated matrix effects could lead to inaccurate reporting (Raposo and Barceló, 2020). Due to the numerous types of matrices cannabis testing laboratories analyze, laboratories are forced to become creative when doing matrix matching for their calibrations. Matrix matching for terpenes in cannabis flower represents a particularly tough issue, because similar plant species to cannabis also contain terpenes. In this study, a novel method of cleaning hops was utilized to provide a terpene-free surrogate for matrix matched calibrations. Matrix blanks were run to demonstrate the cleaned hops were free of terpenes and did not contribute to compound responses for the terpenes of interest (Figure 3). This was the first study to utilize cleaned hops as a clean surrogate for cannabis flower, and the following method validation results demonstrate not only that this was a viable technique, but also it produced a desirable outcome (i.e., excellent method performance).

### Method Performance (DI-SPME Arrow vs. LI-Syringe)

**FIGURE 4:**
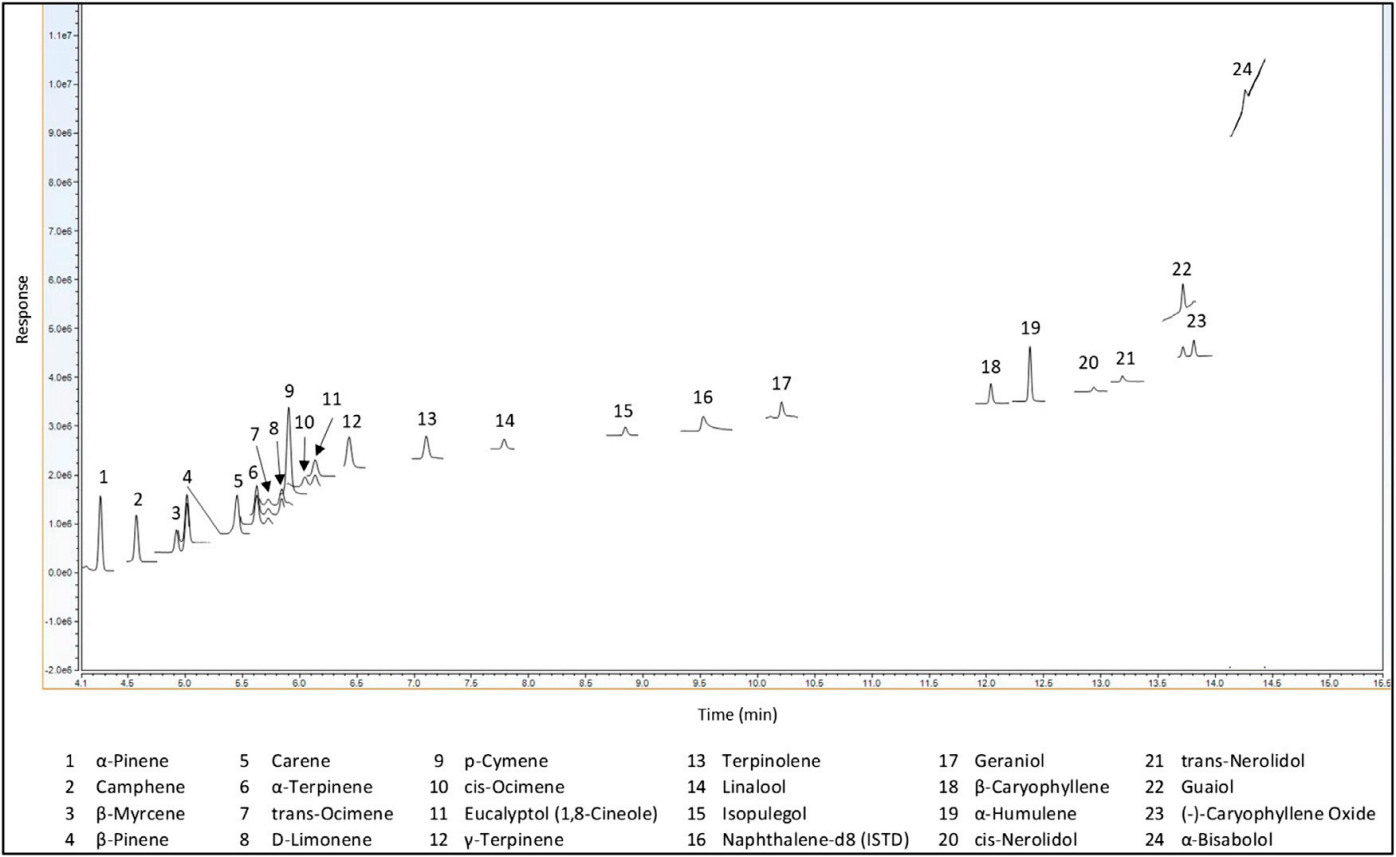
Full separation of 23 terpenes with GC-MS (LI-Syringe).

Method validation was done in accordance with the California Bureau of Cannabis Control guidelines and regulations (California Bureau of Cannabis Control, 2019). Terpenes were analyzed via GC-MS/MS using SIM in single quad MS mode. Single quad MS mode was used to be more representative of most cannabis laboratories, and SIM was used to minimize matrix interferences. All terpenes of interest were resolved using the Rxi-624Sil MS, 30 m × 0.25 mm × 1.4 µm (cat# 13868) as shown in Figure 4.

### DI-SPME Arrow Terpene Validation

Terpene method validation was completed for DI-SPME Arrow to evaluate performance and possible implementation into cannabis testing laboratories. Method performance can be seen in Table 5. As shown in Table 5, an average *r* ^2^ value of 0.991 was achieved for the terpenes of interest in an average working range of 0.08–0.64 μg/mL. α-Pinene had the lowest *r* ^2^ value at 0.965. While most compounds had a working range of 0.08–0.64 μg/mL for calibration, a few compounds (e.g., α-Bisabolol) were able to have expanded ranges from 0.04–0.64 μg/mL and 0.02–0.64 μg/mL. However, two compounds (β-Caryophyllene and α-Humulene) had shortened ranges from 0.08 to 0.32 μg/mL. The differences in calibration working ranges can be attributed to each individual compound’s ability to ad/absorb to the phase on the SPME Arrow and desorb, which can lead to the compound saturation. When compound saturation occurs within the calibration, the calibration curve will plateau, resulting in poor *r* ^2^ values. Previous work looked at higher calibration ranges (>1 μg/mL); however, because the Arrow phase reached its saturation point for this compound class, the calibration range needed to be lowered to a more appropriate linear range. This phenomenon can also be combated by adjusting the parameters (e.g., extraction time, desorption time, etc.), but changing these parameters could then lead to poor results for additional compounds.

**TABLE 5:** DI-SPME Arrow–GC-MS method validation with ASE extracted cleaned hops matrix match for cannabis.

#	Compound	Retention Time (min)	Working Range (μg/mL)	*r* ^2^	LOQ (μg/mL)	Analytical Precision (%RSD)	Method Precision (%RSD)	Recovery (%)
1	α-Pinene	4.475	0.08–0.64	0.965	0.058	7.69	18.5	75.1
2	Camphene	4.813	0.08–0.64	0.993	0.058	7.66	12.2	80.2
3	β-Myrcene	5.184	0.08–0.64	0.983	0.056	7.41	ND	75.7
4	β-Pinene	5.282	0.08–0.64	0.989	0.066	8.78	30.5	90.0
5	Carene	5.746	0.08–0.64	0.983	0.056	7.37	27.1	76.9
6	α-Terpinene	5.937	0.08–0.64	0.996	0.059	7.77	ND	262
7	*trans*-Ocimene	6.042	0.08–0.64	0.995	0.063	8.34	ND	120
8	D-Limonene	6.167	0.08–0.64	0.994	0.062	8.26	ND	78.8
9	*p*-Cymene	6.243	0.08–0.64	0.999	0.060	8.00	6.59	80.1
10	*cis*-Ocimene	6.387	0.08–0.64	0.995	0.069	9.13	35.3	80.3
11	Eucalyptol (1,8-cineole)	6.483	0.04–0.64	0.999	0.031	4.11	7.12	93.1
12	γ-Terpinene	6.780	0.08–0.64	0.999	0.066	8.78	ND	79.9
13	Terpinolene	7.417	0.08–0.64	0.998	0.061	8.03	ND	172
14	Linalool	8.083	0.04–0.64	0.991	0.027	3.60	4.89	93.9
15	Isopulegol	9.109	0.08–0.64	0.993	0.045	5.98	15.5	81.4
16	Naphthalene-d8 (ISTD)	9.765	NA	NA	NA	NA	NA	NA
17	Geraniol	10.429	0.04–0.32	0.996	0.030	4.02	5.46	85.0
18	β-Caryophyllene	12.243	0.08–0.32	0.986	0.073	9.64	33.8	67.2
19	α-Humulene	12.583	0.08–0.32	0.986	0.062	8.23	11.2	71.4
20	*cis*-Nerolidol	13.134	0.04–0.64	0.995	0.038	5.00	10.6	83.6
21	*trans*-Nerolidol	13.382	0.08–0.64	0.993	0.041	5.45	8.36	84.3
22	Guaiol	13.921	0.04–0.64	0.993	0.021	2.83	8.58	88.0
23	(-)-Caryophyllene Oxide	14.024	0.04–0.64	0.994	0.038	4.98	7.85	86.6
24	α-Bisabolol	14.453	0.02–0.64	0.990	0.019	2.58	9.61	90.8
			Average	0.991	0.050	6.68	14.9	95.5

NA, not applicable; ND, not detected.

An average LOQ of 0.050 μg/mL was achieved for the DI-SPME Arrow method. The LOQ was three times the MDL, which was determined off of seven (*n* = 7) replicate injections at 0.08 μg/mL. MDLs were calculated as the standard deviation of the seven replicate measurements multiplied by 3.14 (i.e., the Student’s *t*-value for 99% confidence for seven values). Analytical precision was evaluated using reference standards. Seven replicate injections were made and %RSDs fell below 10% (average = 6.77%) for all terpenes of interest, proving that DI-SPME Arrow has the ability to be a robust analytical technique. In addition to analytical precision, overall method precision (i.e., ASE 350 and DI-SPME Arrow GC-MS) was evaluated by analyzing cannabis shake. Seven shake extractions were made and 1 sample from each extraction was analyzed to evaluate method precision. As seen in Table 5, there is a variance in %RSDs between the different terpenes of interest, and not all terpenes were detected in the shake. Overall, an average %RSD of 14.89% was achieved for the 17 terpenes detected in the cannabis shake. With 14 of the 17 detected terpenes having acceptable %RSDs (i.e., <30%), the remaining 3 terpenes (e.g., β-Caryophyllene) having borderline unacceptable %RSDs. % recoveries ranged from 67.2 to 262% with an average of 95.5%. Three compounds (α-Terpinene, Terpinolene, and β-Caryophyllene) had recoveries outside the acceptable ±30% window and were 262, 172, and 67.2%, respectively.

### Liquid-Injection-Syringe Terpene Validation

Method performance was evaluated for a LI-Syringe method and can be seen in Table 6. Over an average calibration working range of 0.04–5.12 μg/mL, an average *r* ^2^ of 0.993 was achieved for the 23 terpenes of interest. β-Caryophyllene and α-Bisabolol had the smallest calibration range spanning from 0.17 to 5.12 μg/mL, but maintained good *r* ^2^ values of 0.992 and 0.990, respectively. 21 of the 23 terpenes of interest had an *r* ^2^ value of ≥0.990, while the additional two compounds (Geraniol and *trans*-Nerolidol) had an *r* ^2^ value of 0.988. It is important to note that the LI-Syringe technique was able to achieve a higher range for the terpenes.

**TABLE 6:** LI-Syringe–GC-MS method validation with ASE extracted cleaned hops matrix for cannabis.

#	Compound	Retention Time (min)	Working Range (μg/mL)	*r* ^2^	LOQ (μg/mL)	Analytical Precision (%RSD)	Method Precision (%RSD)	Recovery (%)
1	α-Pinene	4.263	0.08–5.12	0.994	0.041	1.36	8.52	93.6
2	Camphene	4.581	0.04–5.12	0.996	0.038	1.25	5.17	93.6
3	β-Myrcene	4.928	0.08–5.12	0.992	0.046	1.51	3.17	91.6
4	β-Pinene	5.020	0.08–5.12	0.998	0.042	1.40	6.20	88.8
5	Carene	5.456	0.08–5.12	0.995	0.057	1.90	4.84	91.2
6	α-Terpinene	5.629	0.04–5.12	0.992	0.036	1.19	5.56	88.0
7	*trans*-Ocimene	5.736	0.04–5.12	0.995	0.022	0.71	*3.35*	88.8
8	D-Limonene	5.851	0.04–5.12	0.994	0.027	0.91	5.25	88.8
9	*p*-Cymene	5.908	0.04–5.12	0.994	0.040	1.32	NA	88.9
10	*cis*-Ocimene	6.053	0.02–5.12	0.993	0.017	0.55	3.04	88.4
11	Eucalyptol (1,8-cineole)	6.142	0.04–5.12	0.996	0.032	1.07	9.04	91.2
12	γ-Terpinene	6.440	0.04–5.12	0.993	0.033	1.09	6.54	88.7
13	Terpinolene	7.112	0.04–5.12	0.993	0.035	1.16	4.35	90.1
14	Linalool	7.795	0.08–5.12	0.991	0.076	2.53	2.99	90.3
15	Isopulegol	8.848	0.04–5.12	0.993	0.029	1.95	3.31	90.2
16	Naphthalene-d8 (ISTD)	9.531	NA	NA	NA	NA	NA	NA
17	Geraniol	10.218	0.04–5.12	0.988	0.039	1.29	3.31	88.2
18	β-Caryophyllene	12.042	0.017–5.12	0.992	0.129	4.27	3.90	91.6
19	α-Humulene	12.384	0.04–5.12	0.991	0.027	0.91	3.88	86.7
20	*cis*-Nerolidol	12.940	0.08–5.12	0.994	0.080	2.67	14.6	89.3
21	*trans*-Nerolidol	13.192	0.08–5.12	0.989	0.050	1.65	2.58	98.9
22	Guaiol	13.720	0.08–5.12	0.993	0.060	1.98	3.27	85.9
23	(-)-Caryophyllene Oxide	13.816	0.04–5.12	0.993	0.030	0.98	1.73	84.6
24	α-Bisabolol	14.296	0.017–5.12	0.990	0.094	3.11	4.84	96.1
			Average	0.993	0.047	1.56	4.94	90.2

NA, not applicable; ND, not detected.

An average LOQ of 0.047 μg/mL was achieved for the LISyringe method, which is nearly identical to the average LOQ achieved for the DI-SPME Arrow method. LOQs for the LISyringe method were calculated identically to what was completed to those for the DI-SPME Arrow method. It should be noted that while the LI-Syringe method had lower LOQs for the more volatile terpenes of interest, the DI-SPME Arrow method had lower LOQs for the majority of the less volatile terpenes of interest. Analytical precision had an average %RSD of 1.62% with all compounds below 5%. Improvements were made when looking at method precision via LI-Syringe vs. DI-SPME Arrow. All terpenes of interest had %RSDs of less than 10%, with the exception of cis-nerolidol (<15%) for method precision. Average %RSD for LI-Syringe was 4.75%. % recoveries ranged from 84.6 to 98.9% with an average of 90.2%. All 23 terpenes of interest fell into the acceptable ±30% window for the LI-Syringe method.

The aforementioned results are comparable to a previously published study by Ibrahim et al. (2019). Ibrahim et al. showed *r* ^2^ value of >0.99, %RSDs <15%, recoveries ranging from 67 to 105.7%, MDLs of 0.25 μg/mL, and LOQs of 0.75 μg/mL. The current study was able to build off of the foundation from Ibrahim et al. and achieved similar *r* ^2^ values, %RSDs, and recoveries. However, this study excelled with lower working calibration range and an order of magnitude lower LOQs (0.047 μg/mL average). In addition, this study employed the use of accelerated solvent extraction and matrix matched calibrations.

### Terpene Chemovar Evaluation With DI-SPME Arrow and LI-Syringe

After method validation, the chemical variety (chemovar) of three different cannabis chemovars was evaluated via ASE and both DI-SPME Arrow and LI-Syringe techniques. Results shown in Table 7 represent the concentration in the sample for Mint Chocolate Chip (i.e., corrected for ASE extraction and dilution). It is important to note that initially three different chemovars (*n* = 9 total) were evaluated by both DI-SPME Arrow and LI-Syringe; however, due to SPME Arrow saturation on the first two chemovars, the Mint Chocolate Chip chemovar underwent a secondary 10× dilution of the cannabis extract. From the data collected, several points should be discussed when comparing the two methods. For the DI-SPME Arrow method, 17 of 23 terpenes were identified and 22 of 23 were identified via LI-Syringe. It should be noted that 1 compound (*p*-Cymene) was not detected (ND) by either technique and should be considered as not present in this chemovar. The DI-SPME Arrow method had several compounds falling outside of the working ranges and several compounds that were ND. It is hypothesized that perhaps the SPME Arrow used for this experiment was reaching the end of its viable lifetime and therefore was not as efficiently adsorbing and/or releasing the terpenes; however, this was not determined. Regardless, the LI-Syringe appeared to provide better results when analyzing a true sample. Nearly all compounds fell within the LI-Syringe calibration working ranges and saturation was not an issue.

**TABLE 7:** Mint Chocolate Chip terpene content determined by DI-SPME Arrow and LI-Syringe.

#	Compound	DI-SPME Arrow (μg/g)	LI-Syringe (μg/g)
1	α-Pinene^a^	61.2	32.2
2	Camphene	3.22	3.84
3	β-Myrcene	5.17	8.24
4	β-Pinene	35.2	25.7
5	Carene^b^	ND	1.59
6	α-Terpinene	ND	1.95
7	*trans*-Ocimene^a^	0.63	2.02
8	D-Limonene^a^	52.7	52.1
9	*p*-Cymene	ND	ND
10	*cis*-Ocimene^a^	0.36	4.55
11	Eucalyptol (1,8-cineole)	0.56	1.46
12	γ-Terpinene	ND	1.97
13	Terpinolene	ND	2.21
14	Linalool^a^	97.5	106
15	Isopulegol	2.59	3.96
16	Naphthalene-d8 (ISTD)	NA	NA
17	Geraniol	3.04	4.45
18	β-Caryophyllene^a^	17.4	45.6
19	α-Humulene	3.47	14.8
20	*cis*-Nerolidol	1.86	25.4
21	*trans*-Nerolidol	11.3	9.46
22	Guaiol^b^	ND	1.61
23	(-)-Caryophyllene Oxide	7.27	2.97
24	α-Bisabolol	7.97	14.7
	Average	18.3	16.7

^a^ Concentration outside of calibration range for DI-SPME Arrow.

^b^ Concentration outside of calibration range for LI-Syringe.

**FIGURE 5:**
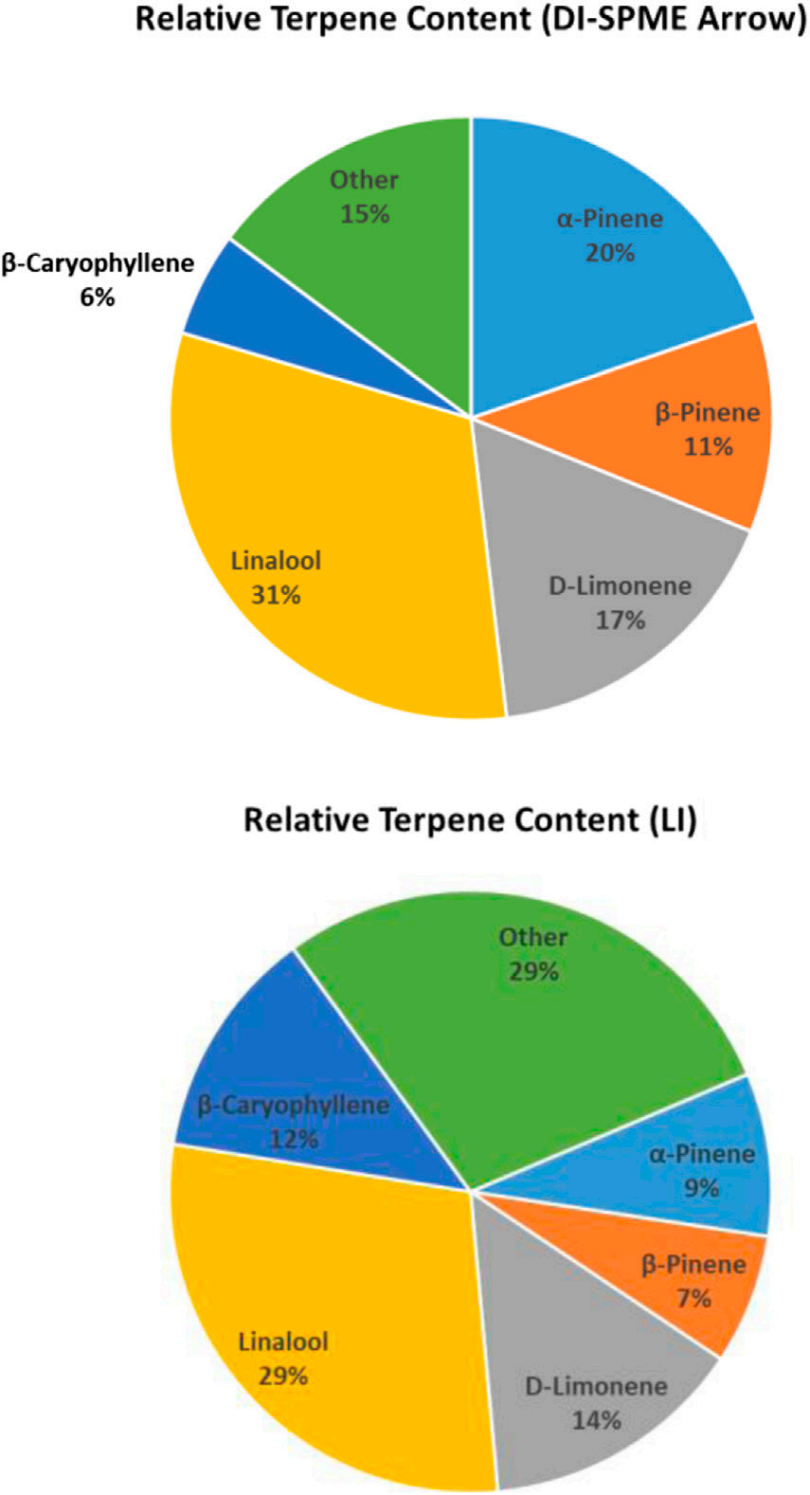
Mint Chocolate Chip terpene profiles determined by DI-SPME Arrow and LI-Syringe.

Despite the aforementioned discrepancies, the average concentration for DI-SPME Arrow was 18.3 μg/g, while the LI-Syringe average concentration was 16.7 μg/g. Comparing the average concentrations specifically, both techniques showed similar concentrations for half of the compounds and divergent concentrations for the other half. More importantly, when comparing the overall terpene profiles of each injection technique, the profiles showed similar results (Figure 5). In particular, both DI-SPME Arrow and LI-Syringe shared the same top five most abundant compounds, which include α-Pinene, β-Pinene, D-Limonene, Linalool, and β-Caryophyllene. Granted, α-Pinene’s response was almost 2× greater for DI-SPME Arrow, while β-Caryophyllene’s response was over 2.5× greater for LI-Syringe. Alternatively, the percentages for D-Limonene and Linalool fell within 3% of one another. Note that the other 18 terpenes were summed in the “other” category. The LI-Syringe had more positive hits for lower concentration terpenes, as its calibration range was lower than the DI-SPME Arrow, hence having the 29% “other” for LI-Syringe vs. the 15% “other” for DI-SPME Arrow.

## Conclusions, Future Work, and Recommendations

The current study demonstrated that DI-SPME Arrow performed better than HS-SPME Arrow; however, both of these approaches outperformed HS-Syringe for the extraction and analyses of terpenes. A novel method for cleaning hops was developed to provide terpene-free hops utilized as a surrogate for matrix matched calibrations. For the first time, both hops and cannabis flower were extracted with ASE and then utilized for method validation.

Validated methodologies for analyzing terpenes in cannabis flower were developed for both DI-SPME Arrow and Liquid-Injection- (LI-) Syringe with GC-MS/MS in single quad mode and selected ion monitoring (SIM). Both methods proved to be viable options for the analysis of terpenes in cannabis flower. When comparing the average LOQs of the terpenes of interest, both techniques were near identical. However, results suggest that LI-Syringe would be the preferred approach for this analysis based on several observations. The LI-Syringe showed an expanded working calibration range compared to the DI-SPME Arrow, which can be attributed to phase saturation of the SPME Arrow. In addition, better analytical and method precision was achieved by LI-Syringe. Furthermore, LI-Syringe appeared to provide a more complete chemovar profile of cannabis flower and at higher concentrations. Lastly, SPME Arrows needed routine replacement due to phase swelling and/or lifetime; a fate which was not shared by LI-Syringe.

While results indicate that LI-Syringe is the preferred technique, other factors should be considered for future work. When running LI-Syringe, more GC maintenance may be needed when compared to the popular HS methods in the cannabis testing industry. This includes inlet consumable changes, analytical column trimming, and MS maintenance, which will come as a result of the matrix being injected into the system. One area not explored in the current work that should be evaluated in future work is instrument uptime for DI-SPME Arrow vs. LI-Syringe. It is hypothesized that less matrix may be exposed to the GC when running the DI-SPME Arrow method, which in turn may lead to longer instrument uptime and less time and money spent on maintenance.

It is recommended that the scientific cannabis community reconsider utilizing HS-Syringe for the analysis of terpenes in cannabis products, as the current results suggest it is inferior to all of the approaches discussed, especially LI-Syringe. Therefore, it is also recommend that the Full Evaporative Technique (FET), which is a HS-Syringe-based approach, also be reassessed in the future. Furthermore, the FET approach is not amenable to splitting samples for other cannabis test methods (e.g., potency), like the ASE method outlined in this study. In addition, it is recommended that future cannabis work continue to evaluate the use of terpene-free hops for surrogate matrix matching of flower. Furthermore, it is recommended that additional cannabis research further the development of ASE methods for the extraction and analysis of terpenes. Under the appropriate conditions, the ASE has the potential to improve laboratory workflows by using one extraction and splitting that extract between multiple analyses in addition to terpene profiling (e.g., potency, pesticides, and mycotoxins). Lastly, it is recommended that this work should be expanded to additional matrices that cannabis testing laboratories frequently analyze (e.g., shatters and waxes), and expand upon the cannabis ME knowledge, which has only begun here. These additional matrices bring new challenges and will need to be addressed to improve the science of cannabis testing.

## Data Availability

The original contributions presented in the study are included in the article/Supplementary Material; further inquiries can be directed to the corresponding author.
