# Supplementary material for: Accelerated Solvent Extraction of Terpenes in Cannabis Coupled With Various Injection Techniques for GC-MS Analysis
Source: Front Chem. 2021 Apr 1;9:619770. doi: 10.3389/fchem.2021.619770 (PMC8047638; doi:10.3389/fchem.2021.619770)
Supplement: Supplementary file 1 [file datasheet1.pdf]

# Accelerated Solvent Extraction of Terpenes in Cannabis Coupled with Various Injection Techniques for GC-MS Analysis

Myers, Colton[1]; Herrington, Jason S.[1]; Hamrah, Paul[2]; Anderson, Kelsey[2]

1. Restek Corporation, 110 Benner Circle, Bellefonte, PA 16823, United States
2. Verity Analytics, 8888 Miramar Road, San Diego, CA 92126, United States

## *Supplementary Material*

Table S1 - Analytical parameters for evaluating terpenes in hops with HS-Syringe, HS-SPME Arrow, and DI-SPME Arrow.

| **Agilent 7890B/5977B GC-MS Parameters** | |
|---|---|
| **Column** | Rxi-624Sil MS – 30m x 0.25mm x 1.4µm (cat# 13868) |
| | |
| **Injection** | HS-Syringe in **Error! Reference source not found.**. HS-SPME Arrow and DI-SPME Arrow in **Error! Reference source not found.** |
| Mode | Split (50:1) |
| | |
| Liner | Topaz 1.8 mm ID Straight/SPME Inlet Liner (cat# 23280) |
| Inj. Temp. | 280 °C |
| Purge Flow | 3 mL/min |
| | |
| **Oven** | 55 °C (hold 0 min) to 130 °C (hold 6 min) by 25 °C/min to 280 °C (hold 1 min) by 30 °C/min |
| | |
| **Carrier Gas** | He, constant flow |
| Flow Rate | 2.0 mL/min |
| | |
| **Detector** | HES - MS |
| Mode | Scan |
| Transfer Line Temp. | 280 °C |
| Source Temp. | 350 °C |
| Quad Temp. | 200 °C |
| Acquisition Range | 35 - 350 amu |
| Rate | 8.2 scans/sec |
| Solvent Delay | 3.00 min |

Table S2 - HS-Syringe parameters for terpenes introduction to GC-MS.

| **CTC PAL Parameters** | |
|---|---|
| **Tool** | HS-Syringe |
| **Syringe Temperature** | 150 °C |
| **Agitator** | Agitator 1 |
| **Injector Penetration Depth** | 40 mm |
| **Incubation Time** | 30 min |
| **Incubation Temperature** | 80 °C |
| **Purge** | 90 s |

Table S3 - HS-SPME Arrow and DI-SPME Arrow parameters for terpenes introduction to GC-MS.

| **CTC PAL Parameters** | |
|---|---|
| **Tool** | HS-SPME / DI-SPME |
| **Agitator** | Agitator 1 |
| **Heatex Stirrer** | Heatex Stirrer 1 |
| **Injector Penetration Depth** | 50 mm |
| **Incubation Time** | 2 min |
| **Extraction Time** | 4 min |
| **Incubation / Extraction Temperature** | 40 °C |
| **Desorption Time** | 60 s |
| **Pre / Post Conditioning** | Yes / No |
| **Conditioning Time** | 60 s |
| **Conditioning Temperature** | 280 °C |

Table S4 - MS SIM Parameters.

| # | Name | Retention Time (min) | Masses |
|---|---|---|---|
| 1 | α-Pinene | 4.52 | 91, 92.1, 93.1 |
| 2 | Camphene | 4.88 | 79.1, 93.1, 121 |
| 3 | β-Myrcene | 5.2 | 93.1 |
| 4 | β-Pinene | 5.39 | 79.1, 93.1 |
| 5 | Carene | 5.81 | 77, 93.1 |
| 6 | α-Terpinene | 5.99 | 77, 93.1 |
| 7 | *trans*-Ocimene | 6.12 | 93.1 |
| 8 | D-Limonene | 6.23 | 93.1 |
| 9 | *p*-Cymene | 6.33 | 119 |
| 10 | *cis*-Ocimene | 6.44 | 93.1 |
| 11 | Eucalyptol (1,8-Cineole) | 6.57 | 81 |
| 12 | γ-Terpinene | 6.87 | 91, 93.1 |
| 13 | Terpinolene | 7.49 | 91, 93.1 |
| 14 | Linalool | 8.16 | 91. 92.1, 93.1 |

| | | | |
|---|---|---|---|
| 15 | Isopulegol | 9.19 | 67, 69.1 |
| 16 | Naphthalene-d8 (ISTD) | 9.83 | 136 |
| 17 | Geraniol | 10.51 | 67.1, 69.1 |
| 18 | β-Caryophyllene | 12.19 | 91, 93.1 |
| 19 | α-Humulene | 12.56 | 91, 92.1, 93.1 |
| 20 | *cis*-Nerolidol | 13.15 | 79.1, 91, 93.1 |
| 21 | *trans*-Nerolidol | 13.42 | 79.1, 91, 93.1 |
| 22 | Guaiol | 13.95 | 93.1, 161 |
| 23 | (-)-Caryophyllene Oxide | 14.12 | 69.1, 79.1, 93.1 |
| 24 | α-Bisabolol | 14.52 | 93.1, 119 |

Table S5 - Terpene calibration levels for DI-SPME Arrow (post ASE extraction).

| **Calibration Curve Serial Dilution Procedure** | | | | |
|---|---|---|---|---|
| **Cal Level** | **Concentration (µg/mL )** | **Amount of previous standard added (µL )** | **Amount of IPA added (µL )** | **Final Volume (mL)** |
| **7** | 12.8 | -- | -- | 3 |
| **6** | 6.4 | 1500 | 1500 | 3 |
| **5** | 3.2 | 1500 | 1500 | 3 |
| **4** | 1.6 | 1500 | 1500 | 3 |
| **3** | 0.8 | 1500 | 1500 | 3 |
| **2** | 0.4 | 1500 | 1500 | 3 |
| **1** | 0.2 | 1500 | 1500 | 3 |

Table S6 - Terpene calibration levels for DI-SPME Arrow (post dilution in headspace vial).

| **Calibrator Concentration at Instrument** | | | | |
|---|---|---|---|---|
| **Cal Level** | **Concentration (µg/mL )** | **Concentration at Instrument (µg/mL )** | **Amount of previous calibrator added (mL)** | **Final Volume (mL)** |
| **7** | 12.8 | 0.64 | 1 | 20 |
| **6** | 6.4 | 0.32 | 1 | 20 |
| **5** | 3.2 | 0.16 | 1 | 20 |
| **4** | 1.6 | 0.08 | 1 | 20 |
| **3** | 0.8 | 0.04 | 1 | 20 |
| **2** | 0.4 | 0.02 | 1 | 20 |
| **1** | 0.2 | 0.01 | 1 | 20 |

Table S7 - Terpene calibration levels for LI-Syringe (post ASE extraction and dilution).

| Cal Level | Concentration (µg/mL ) | Amount of previous standard (µL) | Amount of IPA (µL) | Final Amount (µL ) |
|---|---|---|---|---|
| **10** | 5.12 | -- | -- | 500 |
| **9** | 2.56 | 500 | 500 | 500 |
| **8** | 1.28 | 500 | 500 | 500 |
| **7** | 0.64 | 500 | 500 | 500 |
| **6** | 0.32 | 500 | 500 | 500 |
| **5** | 0.16 | 500 | 500 | 500 |
| **4** | 0.08 | 500 | 500 | 500 |
| **3** | 0.04 | 500 | 500 | 500 |
| **2** | 0.02 | 500 | 500 | 500 |
| **1** | 0.01 | 500 | 500 | 1000 |
