# Supplementary material for: Accelerated Solvent Extraction of Terpenes in Cannabis Coupled With Various Injection Techniques for GC-MS Analysis
Source: Front Chem. 2021 Apr 1;9:619770. doi: 10.3389/fchem.2021.619770 (PMC8047638; doi:10.3389/fchem.2021.619770)
Supplement: Supplementary file 2 [file datasheet2.pdf]

Accelerated Solvent Extraction of Terpenes in Cannabis Coupled with Various Injection Techniques for GC-MS Analysis

Myers, Colton[1]; Herrington, Jason S.[1]; Hamrah, Paul[2]; Anderson, Kelsey[2]

1. Restek Corporation, 110 Benner Circle, Bellefonte, PA 16823, United States
2. Verity Analytics, 8888 Miramar Road, San Diego, CA 92126, United States

Supplementary Material

Table S1 - Analytical parameters for evaluating terpenes in hops with HS-Syringe, HS-SPME Arrow, and DI-SPME Arrow.

**Agilent 7890B/5977B GC-MS Parameters**	
**Column**	Rxi-624Sil MS – 30m x 0.25mm x 1.4µm (cat# 13868)
**Injection**	HS-Syringe in Table S2. HS-SPME Arrow and DI-SPME Arrow in Table S3
Mode	Split (50:1)
Liner	Topaz 1.8 mm ID Straight/SPME Inlet Liner (cat# 23280)
Inj. Temp.	280 °C
Purge Flow	3 mL/min
**Oven**	55 °C (hold 0 min) to 130 °C (hold 6 min) by 25 °C/min to 280 °C (hold 1 min) by 30 °C/min
**Carrier Gas**	He, constant flow
Flow Rate	2.0 mL/min
**Detector**	HES - MS
Mode	Scan
Transfer Line Temp.	280 °C
Source Temp.	350 °C
Quad Temp.	200 °C
Acquisition Range	35 - 350 amu
Rate	8.2 scans/sec
Solvent Delay	3.00 min

Table S2 - HS-Syringe parameters for terpenes introduction to GC-MS.

**CTC PAL Parameters**	
**Tool**	HS-Syringe
**Syringe Temperature**	150 °C
**Agitator**	Agitator 1
**Injector Penetration Depth**	40 mm
**Incubation Time**	30 min
**Incubation Temperature**	80 °C
**Purge**	90 s

Table S3 - HS-SPME Arrow and DI-SPME Arrow parameters for terpenes introduction to GC-MS.

**CTC PAL Parameters**	
**Tool**	HS-SPME / DI-SPME
**Agitator**	Agitator 1
**Heatex Stirrer**	Heatex Stirrer 1
**Injector Penetration Depth**	50 mm
**Incubation Time**	2 min
**Extraction Time**	4 min
**Incubation / Extraction Temperature**	40 °C
**Desorption Time**	60 s
**Pre / Post Conditioning**	Yes / No
**Conditioning Time**	60 s
**Conditioning Temperature**	280 °C

Table S4 - MS SIM Parameters.

#	Name	Retention Time (min)	Masses
1	α-Pinene	4.52	91, 92.1, 93.1
2	Camphene	4.88	79.1, 93.1, 121
3	β-Myrcene	5.2	93.1
4	β-Pinene	5.39	79.1, 93.1
5	Carene	5.81	77, 93.1
6	α-Terpinene	5.99	77, 93.1
7	*trans*-Ocimene	6.12	93.1
8	D-Limonene	6.23	93.1
9	*p*-Cymene	6.33	119
10	*cis*-Ocimene	6.44	93.1
11	Eucalyptol (1,8-Cineole)	6.57	81
12	γ-Terpinene	6.87	91, 93.1
13	Terpinolene	7.49	91, 93.1
14	Linalool	8.16	91. 92.1, 93.1
15	Isopulegol	9.19	67, 69.1

16	Naphthalene-d8 (ISTD)	9.83	136
17	Geraniol	10.51	67.1, 69.1
18	β-Caryophyllene	12.19	91, 93.1
19	α-Humulene	12.56	91, 92.1, 93.1
20	*cis*-Nerolidol	13.15	79.1, 91, 93.1
21	*trans*-Nerolidol	13.42	79.1, 91, 93.1
22	Guaiol	13.95	93.1, 161
23	(-)-Caryophyllene Oxide	14.12	69.1, 79.1, 93.1
24	α-Bisabolol	14.52	93.1, 119

Table S5 - Terpene calibration levels for DI-SPME Arrow (post ASE extraction).

Cal Level	Concentration (µg/mL)	Amount of previous standard added (µL)	Amount of IPA added (µL)	Final Volume (mL)
**7**	12.8	NA	NA	NA
**6**	6.4	1500	1500	3
**5**	3.2	1500	1500	3
**4**	1.6	1500	1500	3
**3**	0.8	1500	1500	3
**2**	0.4	1500	1500	3
**1**	0.2	1500	1500	3

NA – Not Applicable

Table S6 - Terpene calibration levels for DI-SPME Arrow (post dilution in headspace vial).

Cal Level	Concentration (µg/mL)	Amount calibrator added (mL)	Amount of water added (mL)	Final Volume (mL)	Concentration at Instrument (µg/mL)
**7**	12.8	1	19	20	0.64
**6**	6.4	1	19	20	0.32
**5**	3.2	1	19	20	0.16
**4**	1.6	1	19	20	0.08
**3**	0.8	1	19	20	0.04
**2**	0.4	1	19	20	0.02
**1**	0.2	1	19	20	0.01

Table S7 - Terpene calibration levels for LI-Syringe (post ASE extraction and dilution).

Cal Level	Concentration (µg/mL)	Amount of previous standard (µL)	Amount of IPA (µL)	Final Amount (µL)
**10**	5.12	NA	NA	NA
**9**	2.56	500	500	500
**8**	1.28	500	500	500
**7**	0.64	500	500	500
**6**	0.32	500	500	500
**5**	0.16	500	500	500
**4**	0.08	500	500	500
**3**	0.04	500	500	500
**2**	0.02	500	500	500
**1**	0.01	500	500	1000

NA – Not Applicable
